# Text analysis of billboards and infographic graphics advertising COVID-19 on promoting preventive behaviors and taking vaccination against the coronavirus pandemic and investigating the opinions of the Iranian adult population

**DOI:** 10.1186/s12889-024-18135-3

**Published:** 2024-03-01

**Authors:** Fereshteh Mohamadpour, Gary Groot, Ardalan Askarian, Mehrdad Askarian

**Affiliations:** 1 Department of Community Medicine, School of Medicine, Shiraz University of Medical Sciences, Shiraz, Iran; 2 Department of Community Health and Epidemiology, University of Saskatchewan, Saskatoon, Canada; 3 College of Arts & Science, University of Saskatchewan, Saskatoon, Canada

**Keywords:** Coronavirus, Communication, Mass media, Disease prevention, Medical education

## Abstract

**Background:**

Advertising is one of the most important solutions that health centers and medical services around the world use to try to encourage public opinion to create a positive attitude towards preventive measures and vaccination. This study has been done with the aim of text analysis of billboards and infographics related to promoting preventive behaviors and vaccination against the coronavirus pandemic and providing solutions and models for preventive information and advertising in the field of health.

**Methods:**

The study method in this research is a combination of qualitative and content analysis. Data collection was done in a targeted manner. The sample size includes 33 advertising billboards and infographics. Data collection has been done through searching the sites and websites of health networks and medical education centers in Iran, taking pictures of infographics and billboards in public places, and also receiving archive files of pictures from the public relations of health networks and medical services. The data was collected from February 19, 2020 to December 30, 2022 (the time frame of the pandemic and public vaccination program in Iran). The data was analyzed based on the three-dimensional discourse analysis theory of Fairclough. Then, an online survey about promoting preventive behaviors and vaccination against the coronavirus pandemic in the format of billboards and infographics was designed in SurveyMonkey and its link was provided to the audience through virtual networks and other platforms. The age group of people was selected from 18 to 70 years. Considering that the number of participants should be representative of the entire community under investigation, therefore, based on Cochran’s formula, the sample size was equal to 350 people. Finally, users’ opinions were analyzed using descriptive statistics. The assessment of validity involved experts in infection control and linguistics. The reliability of the measurement, determined through the Cronbach’s alpha internal consistency coefficient, yielded a coefficient of 0.968.

**Results:**

The results show that among the four linguistic components of words, syntax, coherence and text structure; “live metaphors”, “pronoun “we”, “collocation and reference”, and “attitude markers” have the most impact on the audience. The frequency percentage of the data shows that these language elements have tremendous power in attracting the audience to perform preventive behaviors. The results show that the language reflects the culture, opinions and needs of people in the society. Also, the results show that encouraging people to perform preventive behaviors should be through the integration of medical information with motivational linguistic factors in order to attract the audience more.

**Conclusions:**

It can be concluded that the use of the appropriate pattern of medical advertising discourse and correct communication strategies, will help public participation in the field of epidemic control. The language of effective health education and health communication during an epidemic must be related to the ways of thinking and speaking of ordinary people. Also, words with metaphorical and ironic meanings have a high potential to influence the health performance of people in society and increase public awareness of health communication. Therefore, using them to create a new value system with the aim of controlling and overcoming the consequences of the epidemic is very effective.

## Background

Infectious diseases have existed throughout history and have intensified over time. In recent times, even new infectious diseases have appeared, the speed of which the speed of their progress is increasing in the world, and they have created a significant impact on global health [1]. Corona Virus is a new infectious disease that is transmitted through respiratory droplets and contact [2]. This disease was one of the main causes of death, disability, and social and economic problems for millions of people [3] creating a very difficult situation for people all over the world [4].

Preventive measures in the healthcare sector, especially now, are extremely important [5, 6]. Organizations in charge of health in different countries have always sought the goal that all people have the possibility of a healthy and quality life and to reduce the gap between the quality of services through health service training and preventive measures [7]. Empowering people to increase control over their own health and continuously improving the health of the society they live in will promote health [8]. Health promotion includes behaviors and actions through which people will have more control over decisions and actions that affect their health and that of their community [9]. Increasing people’s awareness of the causes and consequences of diseases has a significant contribution in promoting preventive behaviors. Advertising is one of the most convenient, cost-effective and effective strategies that health centers and medical services around the world use to try to influence public opinion to create a positive attitude towards preventive measures and vaccination.

So far, researchers have conducted research in various fields regarding the ways to prevent and control the Corona Virus. These researches include topics such as epidemiological research [10, 11], Disease Prevention [12–15], diagnostic methods [16–18], clinical characteristics of the disease [19, 20], characteristics of disease transmission [21, 22], development of candidate therapies [23–25]. In recent decades, researchers have become increasingly interested in research that investigates the role of language in creating surrounding realities. The set of social and cultural conditions creates the context for the occurrence of text or writing, speech and non-verbal communication in a general proposition [26]. Therefore, language is a repository of human knowledge about the outside world and a systematic set of meaningful categories that help humans face new experiences and store information about previous experiences [27]. Few research has been done in the field of linguistic analysis on the topic of Corona epidemic. These research include topics such as Linguistic and meta-linguistic meanings of words related to the corona virus [28], the effects of the corona virus and the social crisis caused by it, as well as neologisms on language changes [29], linguistic analysis of fear-factor lexemes on Coronavirus Pandemic [30], reflection on the social and psychological consequences of the Coronavirus Pandemic in the new vocabulary of medical discourse [31], content analysis of communication strategies and their effects on public engagement on social media [32] and hesitancy from Covid-19 Vaccination [33].

In a society where people witnessed the explosion of information on the Internet and other media, the existence of various information about Corona (prevention and general vaccination) caused confusion or even created false and incorrect thoughts in the society [34–36]. For this reason, people’s attention was mainly drawn to official government announcements around the world, as well as official opinions of the medical community, so that they could receive reliable information [37]. Infographics and billboards were among the most important tools that all health networks and medical services around the world used during the Corona epidemic to increase the level of public awareness so that they could manage the existing crisis.

Although many studies have been conducted on various aspects of the epidemic and its impact on people’s lives, but still, the linguistic dimensions and the analysis of the discourse structures around the corona as a main element in expressing and inducing the concepts related to this crisis at the level of the society have not been discussed and investigated seriously. Also, humans have special procedures to give meaning to their daily life. In this regard, language is considered as a relationship or mediator of the social world, a very important element. It is necessary and useful to pay scientific and careful attention to the issue of communication with people in the society using different forms of language at micro and macro levels in order to analyze and explain some issues related to medical science. Every person uses certain types of language in different situations of discourse and according to the type of culture, language, social norms, gender, age, job, etc. Discourse situations form specific discourse processes. Conversation in institutional or organizational settings, including therapeutic and disease prevention situations, requires rules not typically found in personal situations. The role that linguistic agents and discourses play in these specific situations will influence the possible topics discussed as well as the interpretation provided about people’s social actions. This is also extremely important in medical researches. Therefore, the current research aims to investigate the discourse related to the Corona pandemic from a different angle with an interdisciplinary perspective that is a combination of medical science and linguistics. Providing information in the form of billboards and infographics (multimedia contents) reduces the time it takes for the audience to receive information, and it is much more effective and usable than just written-verbal messages. Considering that the presentation of multimedia contents in informing people is more general, therefore, when producing such contents by medical service centers, it is very important to consider the basic culture of each society. Considering the mentioned points and that the target population in Iran is all strata of the society; therefore, the results of the current research, in addition to promoting preventive behaviors and vaccination of the corona pandemic, are used as a more general model to promote the improvement of the health culture of the society. Among the other achievements of the results of this research is the presentation of a final model of multimedia content based on Iranian culture with maximum effectiveness on the general population of Iran which can be used to train the health personnel in similar possible crises as well as the professional and planned control and management of such crises.

## Methods

The method used in this research is qualitative content analysis. For content analysis, Fairclough’s three-dimensional discourse analysis theory [38] has been used. Compared to other theories, this theory is one of the most documented theories for research in the field of communication, culture and society. Because compared to similar theories such as Iedema’s theory [38], Jewitt et al.’s theory [39] and Hull’s theory [40], it is much more comprehensive and has placed textual components, social components and discourse components in the center of analysis [37]. In Fairclough’s model, the four components of language such as “word”, “syntax”, “coherence” and “text structure” are investigated. Based on this model, in each of the mentioned parts, a number of questions are asked, and the answers to these questions lead to achieving a desired result and finally providing a model related to the topic under investigation. In Table 1, explanations related to Fairclough’s theory are given.

**Table 1:** Fairclough’s discourse theory [38]

Linguistic dimensions	Questions
Word	Question 1: What is the empirical value of words?
	Question 2: What communication value do words have?
	Question 3: What does the expressive value of the words in the sentence refer to in the real world?
	Question 4: What metaphors or virtual meanings are used in the sentence?
Syntax	Question 1: What are the empirical values of the grammar used in the text?
	Question 2: What are the communicative values of the grammar used?
	Question 3: What expressive value can be seen in the grammatical characteristics of the sentence?
Coherence	Question 1: How are simple sentences combined with each other?
Text Structure	Question 1: What methods have been used for interaction?
	Question 2: Which part of the text has more emphasis and which part contains the message?

### Data collection

Data collection has been done through searching the sites and websites of health networks and medical education centers in Iran, taking pictures of infographics and billboards in public places, and also receiving archive files of pictures from the public relations of health networks and medical services. The data was collected from February 19, 2020 to December 30, 2022 (the time frame of the pandemic and public vaccination program in Iran). The samples have been purposefully selected. The way of selection and criteria for entering and exiting billboards and infographics in the research is as follows: Billboards and infographics according to the written text of advertisements, color, arrangement of elements, two- or three-dimensionality of images, size of writings, shapes, their locations in the city and factors that affect the audience’s attention. Also, the samples were selected by considering the characteristic of having at least one linguistic factor along with advertising and persuasive slogans to analyze the textual, argumentative and social dimensions. The criterion for choosing the sites from which the billboards and infographics were taken were to be popular and have enough credibility to gain the trust of the audience. For this reason, reliable news sites and public relations sites of scientific centers affiliated to the Ministry of Health, Medicine and Medical Education of Iran were selected as target sites. The method of selection and the entry and exit criteria of the participants, according to the type of subject, tried to select them from different strata of the society and from the group at risk of corona virus as far as possible. In order to achieve this goal, the study field included citizens living in all parts of Iran. The criteria for entering people into this research are: Monolingual, the ability to speak Persian, the ability to read and write, not having jobs and disciplines such as literature, writing and teaching, in which case people are considered professional users of speech and language. Exclusion criteria include unwillingness to voluntarily participate in the survey, lack of honest cooperation of participants, bilingualism or multilingualism, professional language user, inability to read and write. The sample size was the number required to reach theoretical saturation and a new pattern can be extracted from it. In this research, 33 advertising billboards and infographics were selected. Since the current research is qualitative, efforts will be made to cover the maximum variety of samples. After data selection and text analysis, a survey form containing preventive advertising and vaccination texts was designed in SurveyMonkey in order to collect field data and more detailed investigations of the impact of the messages on the audience. Then, the link of the questionnaire was provided to the audience through virtual networks and platforms to investigate and analyze the opinion of the audience about the texts. The statistical population includes all strata of society and from the group at risk of Corona Virus in Iran in the corona and post-corona years. Considering that the number of participants should be representative of the entire community under investigation, therefore, based on Cochran’s formula [39], the sample size was equal to 350 people. The age group of people was selected from 18 to 70 years. After collecting the field data, the texts in the questionnaire were classified and coded based on the linguistic components of Fairclough’s three-dimensional discourse analysis theory. Then, the opinions of the participants in the survey investigated and based on descriptive statistic, the frequency of each linguistic component calculated. Finally, the discourse model designed based on the highest frequency of data. The assessment of validity involved experts in infection control and linguistics. The reliability of the measurement, determined through the Cronbach’s alpha internal consistency coefficient, yielded a coefficient of 0.968.

## Results

The texts were analyzed using Fairclough’s three-dimensional model. Then, advertising texts were distributed in the form of an online questionnaire among the audience in the age group of 18 to 70 years to check the impact of these texts on the audience. Table 2 shows the linguistic data taken from the billboards and infographics of this research.

**Table 2:**
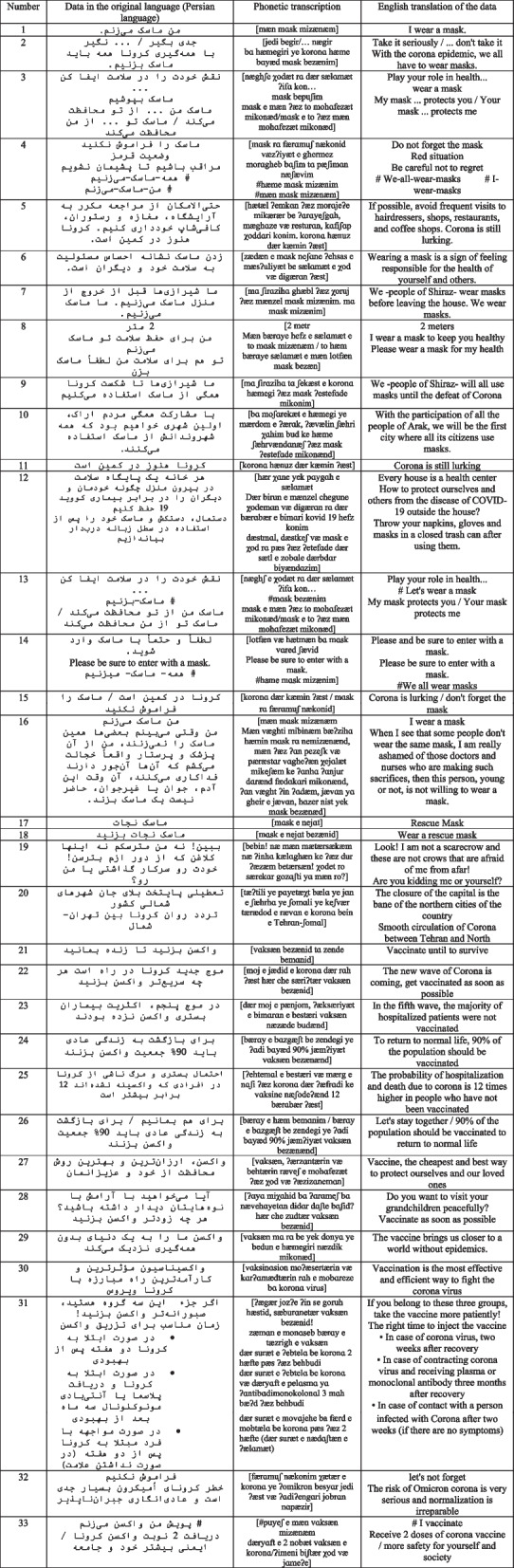
Linguistic data of billboards and infographics

### Qualitative analysis

#### Words analysis

##### Words experimental value

**Classification of words**

Word structure in all languages has a special order and consists of components. The structure of the word in the Persian language consists of components, based on which it is divided into “simple”, “compound”, “derivative”, and “derivative-compound” types. Each of the words has a specific meaning based on the type of structure, and by being placed next to each other and making sentences, they can convey a special meaning that the other type with a different structure, this time will not convey meaning to the audience. Below are the types of words with examples.

*Simple words*

Simple words are words that consist of one component and cannot be divided into smaller components [40].

- Example 1: [**mæn mɑsk mizænæm**]
- Example 2: [**mɑsk rɑ færɑmuʃ nækonid**]

*Compound words*

Words that consist of two or more independent and meaningful parts are compound words [40].

- Example 1: [tæʔtili e pɑyetæχ bælɑy e jɑn e ʃæhrhɑy e ʃomɑli e keʃvær / tærædod e rævɑn e koronɑ bein e **Tehran-ʃomɑl**]
- Example 2: [**dæstmɑl**, **dæstkeʃ** væ mask e χod rɑ pæs ʔæz ʔestefɑdeh dær sætl e zobɑle e **dærbdɑr **biyændɑzim]

*Derivational words*

The words that consist of meaningful and component affix (prefix and suffix) are called derivative words [40].

- Example 1: [ʔehtemɑl e bestæri væ mærg e nɑʃi ʔæz koronɑ dær ʔæfrɑdi ke vɑksine næʃodeʔænd, 12 bærɑbær biʃtær ʔæst]
- Example 2: [dær moj e pænjom, ʔæksæriyæt e bimɑrɑn, vɑksæn næzæde budænd]

*Derivative-compound words*

Derivative-compound words are the result of the combination of derivative and compound words [40].

- Example 1: [vɑksæn mɑ rɑ be yek donyɑ e bedun e hæmegiri næzdik mikonæd]
- Example 2: [χætær e koronɑ e ʔomikron besyɑr jedi ʔæst væ ʔɑdiʔengɑri, jobrɑnnɑpæzir]

**Ideology of words**

Ideology is a systematic collection of beliefs, thoughts and intellectual systems hidden in words and the real and main meaning of the writer or speaker to express something [41]. Ideologies can influence behaviors, decisions, and recommendations [42]. In most languages of the world and especially in the Persian language, words that are highlighted have a certain ideology. The words that the text producer wants to be the center of the audience’s attention and convey the message in the best possible way are usually placed at the beginning of the sentence. In the data of this research, mainly words have been highlighted that were either the most important factor in preventing the spread of the corona virus or encouraging people to and do the same. In examples 1 and 2, masking is highlighted in the sentence as the most important element in preventing the spread of the corona virus. In example 3, the prepositional group is at the beginning of the sentence. The word “ʔævælin” which means “Pioneering in doing something to meet the needs of society” will make that activity last in the audience’s mind and encourage her to do it. In example 4, the author of the text applies a certain ideology by using the superlative adjective. Since the audiences of billboards and infographics are the majority of the middle class of the society, therefore, introducing a solution with great efficiency to prevent and protect the people of the society in a free and safe way, can be effective in attracting the audience.

- Example 1: [zædæn e mɑsk neʃɑne ye ʔehsɑs e mæsʔuliyæt be sælɑmæt e χod væ digærɑn ʔæst]
- Example 2: [mɑsk e nejɑt bezænid]
- Example 3: [bɑ moʃɑrekæt e hæmegi ye mærdom e ʔærɑk, ʔævælin ʃæhri χɑhim bud ke hæme ye ʃæhrvændɑnæʃ ʔz mɑsk ʔestefade mikonænd]
- Example 4: [vɑksæn, ʔærzɑntærin væ behtærin ræveʃ e mohɑfezæt ʔæz χod væ ʔæzizɑnemɑn]

**Synonym and opposite**

Synonymous words are words that have similar and identical meanings in terms of concept, and opposite are words that are semantically and conceptually antonym [43]. Placing synonyms or opposite words in sentences attracts the attention of the audience, and the text producer can use them to persuade and encourage the audience to do or not do something.

- Example 1: [vɑksinɑsion moʔæsertærin væ kɑrɑmædtærin rɑh e mobɑreze bɑ koronɑ virus]
- Example 2: [jedi begir / … nægir bɑ hæmegiri ye koronɑ, hæme bɑyæd mɑsk bezænim]

##### Relational value

*Formal and informal words*

How to choose words to create effective communication between different people in the society is very important [26]. The use of formal phrases and words means the use of ceremonial, skillful, conventional or even traditional words that usually belong to a specific social position and dignity [26]. Therefore, the use of official word indicates the class and social status of people. Especially, the use of respectful words gives the audience a special respect [26]. On the other hand, the use of informal words in conversations means the use of informal and sometimes friendly words, which differ according to the level of intimacy between people, their selection and use [26]. In example 1, the producer of the text is trying to give credibility and respect by choosing formal and polite words when communicating with the audience. In example 2, the content producer has marked the text by using proverbs and using informal words instead of formal ones and using the second person singular pronoun. She/he tried to blame people who did not use the mask properly.

- Example 1: (Formal) [lotfæn væ hætmæn bɑ mɑsk vɑred ʃævid]
- Example 2: (Informal) [bebin! næ mæn mætærsækæm næ ʔinhɑ kælɑghæn ke ʔæz dur ʔæzæm betærsæn!]

##### Expressive value

*Vocabulary conceptual signification*

Words have all kinds of relationships that are visible in the semantic system of the language between concepts that may be independent of each other at first glance, but in fact, they are closely related to each other that sometimes it is not possible to distinguish them simply [43]. For example, it is sometimes possible for a concept to include one or more other concepts. In such a situation, the relation of hyponymy is proposed, which is the relationship between a concept and superordinate concepts [43]. A word whose meaning includes the meaning of other words is an inclusive word, and words that are conceptually under the scope of an inclusive word are called hyponym words. In relation to each other, the hyponym words are considered co-hyponym words [43]. The placement of such words (inclusive and hyponym) next to each other instill a special meaning to the audience, which increases the expressive value of the words. In example 1, “red status” indicates serious danger. This phrase is placed next to the phrases “being careful” and “not regretting” which convey a special expressive value to the audience. In fact, “red status” which is a sign of danger and warning, along with providing a solution and expressing the result of following the solution, which is the improvement of the situation, are a combination of conceptual relationships at the vocabulary level that have increased the expressive value of the word in the audience’s mind. In example 2, combining the words “mask”, “wearing” and “protecting” together has increased the expressive value of the words. In Persian language, the word “mask” is placed next to the word “hitting”, but in this text “mask” is placed together with “wearing” and “protecting”, which makes the word attractive. “Wearing” in Persian language is used for clothing and here “mask” is considered as “clothing” or “armor” that by wearing it, people can protect themselves from danger and play a role as an agent in the field of community health promotion.

- Example 1: [mɑsk rɑ færɑmuʃ nækonid
- **væzʔiyæt e ghermez**
- **morɑgheb bɑʃim** tɑ **pæʃimɑn næʃævim**
- #hæme mɑsk mizænim
- #mæn mɑsk mizænæm]
- Example 2: [næghʃ e χodæt rɑ dær sælɑmæt ʔifɑ kon …
- **mɑsk bepuʃim**
- mɑsk e mæn ʔæz to **mohɑfezæt mikonæd**
- mɑsk e to ʔæz mæn **mohɑfezæt mikonæd**]

##### Metaphor

Metaphor is a type of expression technique that is done by using one word or phrase instead of another word or phrase based on the similarity between them [44].

**Dead metaphor**

A dead metaphor is a word or phrase that was formed by convention in the past and entered the vocabulary list of the language [44]. The use of such metaphors in language is unconscious [44]. In example 1, the phrase “sær e kɑr gozɑʃtæn” means “doing useless behavior or work” which has become a slang term due to repetition and is in the mental vocabulary of Persian speakers.

- Example 1: [bebin! næ mæn mætærsækæm næ ʔinhɑ kælɑghæn ke ʔæz dur ʔæzæm betærsæn! χodet ro **sær e kɑr gozɑʃti** yɑ mæn ro]

**Live metaphor**

A living metaphor is a new metaphor that has not been widely used and has not yet been registered in the vocabulary of the language [44]. In example 1, the personification is used. This literary array is a type of metaphor in which the simile (Corona) is non-human and the characteristic of a human (being lurking) is attributed to it. In fact, Corona is likened to a dangerous enemy that is lurking and waiting for a situation to trap humans.

- Example 1: [**koronɑ **dær **kæmin** ʔæst]

##### Metonymy

Metonymy is the use of the word in its non-real and non-original meaning, provided that there is a link between the real and non-real meaning [44]. One of the metonymy types is mentioning the whole and the will of the part, which is mentioned in the following examples. In example number 1, the whole word “pɑyetæχt” is given, and the meaning is to express the component (Tehran City). The meaning of “ʃomɑl” is also the northern cities of Iran. In example 2, the whole meaning “ʔærɑkihɑ” is mentioned and it means “people of Arak city”.

- Example 1: [tæʔtili e **pɑyetæχt** bælɑ ye jɑn e ʃæhrhɑ ye ʃomɑli ye keʃvær / tærædod e rævɑn e koronɑ bein e Tehrɑn-**ʃomɑl**]
- Example 2: [mɑ **ʔærɑkihɑ** tɑ ʃekæst e koronɑ hæmegi ʔæz mɑsk ʔestefɑde mikonim]

#### Syntax analysis

##### Experimental value

**Syntax processes**

*Scrambling*

It is a common term in sentence structure. Every language has a basic structural composition, which is the basis of the sentence structure of that language. Therefore, every language contains principles and parameters [45]. Some languages exhibit a different arrangement of principles, called a scrambling [46]. From Chomsky’s point of view, scrambling is very common in languages that have free syntactic arrangement (such as: Persian, Russian, German and Turkish languages) [47]. The scrambling includes several types. If the scrambling is at the level of a simple sentence, it is called a scrambling distance short, and if it is beyond a simple sentence, it is called a scrambling distance long [47].

*Scrambling distance short*

- Example 1: [**dær moj e pænjom**, ʔæksæriyæt e bimɑrɑn e bestæri vɑksæn næzæde budænd]

*Scrambling distance long*

- Example 1: [**bɑ moʃɑrekæt e hæmegi e mærdom** e ʔærɑk, ʔævælin ʃæhri χɑhim bud ke hæme ʃæhrvændɑnæʃ ʔæz mɑsk ʔestefɑde mikonænd]

*Structural deletion*

Sometimes a structure is removed under conditions. There are different types of deletion. One of the types of deletion is semantic ellipsis. In this type of deletion, a structure is removed from the sentence so that the audience (reader or listener) understands the meaning of the deleted part from the meaning of the whole sentence [48]. Another type of deletion is verbal ellipsis. In this type of deletion, a structure is removed from the sentence that is present in the preceding or following sentences, and the audience (reader or listener) understands the speaker’s meaning based on it [48].

*Semantic ellipsis*

- Example 1: [hær χɑne yek pɑygɑh e sælɑmæt (**ʔæst**)]
- Example 2: [tæʔtili ye pɑyetæχt, bælɑy e jɑn e ʃæhrhɑ ye ʃomɑli ye keʃvær (**ʔæst**)]

*Verbal ellipsis*

- Example 1: [**jedi** begir / …(**jedi**) nægir]
- Example 2: [χætær e koronɑ ye omikron besyɑr jedi ʔæst væ ʔɑdiʔengɑri, jobrɑnnɑpæzir (**ʔæst**)]

##### Semantic roles

*Agent*

An agent is an entity that performs an action or activity intentionally [49].

- Example: [mæn mɑsk mizænæm]

*Experiencer*

Experiencer is an entity that receives sensory or emotional input [49].

- Example: [næghʃe χodæt rɑ dær sælɑmæt ʔifɑ kon…]

*Stimulus*

A stimulus is an entity that unintentionally evokes sensory feelings [49].

- Example: [**vɑksæn**, ʔærzɑntærin væ behtærin ræveʃ e mohɑfezæt ʔæz χod væ ʔæzizɑnemɑn]

*Theme*

A theme is an action or activity that can be done, but it does not change the state of the activity [49].

- Example: [bærɑy e bɑzgæʃt be zendegi ye ʔɑdi bɑyæd 90% jæmʔiyæt vɑksæn bezænænd]

*Patient*

A Patient is transformative of action and changes its state [49].

- Example: [mɑsk e mæn ʔæz to mohɑfezæt mikonæd/mɑsk e to ʔæz mæn mohɑfezæt mikonæd]

*Instrument*

An instrument is something that is used to perform an action [49].

- Example: [dæstmɑl, dæstkeʃ væ mɑsk e χod rɑ pæs ʔæz ʔetefɑde dær **sætl e zobɑle dærbdɑr** biyændɑzim]

*Location*

Location is a place where an action or activity takes place [49].

- Example: [hætæl ʔemkɑn ʔæz morɑjeʔe mikærær be **ʔɑrɑyeʃgɑh**, **mæghɑze** væ **resturɑn**, **kɑfiʃɑp** χoddɑri konim. koronɑ hænuz dær kæmin ʔæst]

*Direction or goal*

A goal or direction is something towards which an action or activity tends [49].

- Example: [mæn bærɑye hefz e sælɑmæt e to mɑsk mizænæm]

*Recipient*

The receiver of the activity, action or event is called recipient [49].

- Example: [(ʃomɑ) vɑksæn bezænid tɑ zende bemɑnid]

*Source or origin*

A source or origin is where the event or activity originates from its [49].

- Example: [mɑ ʃirɑzihɑ ghæbl ʔæz χoruj ʔæz mænzel mɑsk mizænim. mɑ mɑsk mizænim]

*Tense*

The range of time in which the action occurred is called tense [49].

- Example: [dær suræt e movɑjehe ba færd e mobtælɑ be koronɑ **pæs ʔæz 2 hæfte** (vɑksæn bezænid)]

*Beneficiary*

A beneficiary for whose benefit an action or activity or event occurs [49].

- Example: [vɑksæn **mɑ** rɑ be yek donyɑ ye bedun e hæmegiri næzdik mikonæd]

*Manner*

The method according to which the activity or action is performed [49].

- Example: [vɑksæn, **ʔærzɑntærin** væ **behtærin** ræveʃ e mohɑfezæt ʔæz χod væ ʔæzizɑnemɑn]

*Purpose*

Purpose is the reason for which an action or event is done [49].

- Example: [vɑksinɑsion **moʔæsertærin** væ **kɑrʔɑmædtærin** rɑh e mobɑreze bɑ koronɑ virus]

*Cause*

Something that causes an event or action to occur [49].

- Example: [**bærɑy e bɑzgæʃt be zendegi ye ʔɑdi** bɑyæd 90% jæmʔiyæt vɑksæn bezænænd]

**Topicalization**

Topicalization is the process of moving sentence elements and structures from their unmarked (initial) position to the beginning of the sentence and placing them in the subject position [50]. Therefore, whenever the author wants to convey a more important concept to the reader, she/he moves the structure related to that concept from its original position in the sentence and moves it to the beginning of the sentence in the subject position [50]. Because the subject position is the position where important, new and emphasized information is placed in that place [50]. The construction of unmarked sentences in Persian language is as follows: “subject + object + verb”. In both examples below, the preposition group has moved from its position and moved to the topic position and before the subject of the sentence.

- Example 1: [**dær moj e pænjom**, ʔæksæriyæt e bimɑrɑn e bestæri vɑksæn næzæde budænd]
- Example 2: [**bɑ hæmegiri ye koronɑ** hæme bɑyæd mɑsk bezænim]

**Active and passive verb**

A sentence in which the subject or the doer of the work is known is an active sentence, and a sentence in which the subject or the doer is not known is a passive sentence [51]. No passive sentence was found in the analyzed data.

- Example 1: [**vɑksæn mɑ rɑ be yek donyɑ ye bedun e hæmegiri næzdik mikonæd**] (subject + direct object + indirect object + verb)
- Example 2: [**mɑsk e mæn ʔæz to mohɑfezæt mikonæd**] (subject + direct object + verb)

##### Relational value

**Mood**

A Mood is a form of the verb that determines the opinion of the speaker regarding the definiteness or indefiniteness of the event, and the producer of the text expresses his/her attitude towards the occurrence of the verb by means of it [48]. The verb in Persian language has three moods: subjunctive, imperative and indicative.

*Subjunctive mood*

Subjunctive mood is used when the performance of the verb is not real or the speaker and the writer is not sure of its occurrence and intend to express the possibility, idea, desire, intention, order, request and the like [48].

- Example 1: [bɑ hæmegiri ye koronɑ hæme **bɑyæd** mɑsk bezænim]
- Example 2: [**ʔehtemɑl** e bestæri væ mærg e nɑʃi ʔæz koronɑ dær ʔæfrɑdi ke vɑksine næʃodeʔænd 12 bærɑbær ʔæst]

*Imperative mood*

Imperative mood is a type of verb structure that is used to express order, please, request, warning, guidance and the like [48].

- Example 1: [hær che særiʔtær vɑksæn bezænid]
- Example 2: [næghʃ e χodæt rɑ dær sælɑmæt ʔifɑ kon]

*Indicative mood*

Indicative mood is the most common form in Persian language and is used to express real events or events that the author is sure of happening [48].

- Example 1: [dær moj e pænjom, ʔæksæriyæt e bimɑrɑn e bestæri vɑksæn næzæde budænd]
- Example 2: [vɑksæn mɑ rɑ be yek donyɑ ye bedun e hæmegiri næzdik mikonæd]

**Important characteristics of the relation method**

In order to establish a correct and understandable communication with the audience, speech must follow a series of principles so that the audience can understand and interpret the speaker’s words well [26]. If any of the principles of communication are not observed, the audience cannot have a correct interpretation of the speaker’s speech [26]. The most important solutions and principles that are very useful in this field are the principles of Grice cooperation [26], which are explained below.

*Maxim of quantity*

This solution is based on two principles. One is to give as much information as necessary and the other is to not give more information than is necessary [26].

- Example 1: [lotfæn væ hætmæn bɑ mɑsk vɑred ʃævid]
- Example 2: [vɑksinɑsion moʔæsertærin væ kɑrʔɑmædtærin rɑh e mobɑreze bɑ koronɑ virus]

*Maxim of quality*

Maxim of quality is dedicated to truth and honesty in speech and follows two principles [26]. The first principle, don’t say what you believe to be false. And the second principle, don’t say something about which you don’t have enough evidence [26].

- Example 1: [dær moj e pænjom, ʔæksæriyæt e bimɑrɑn e bestæri vɑksæn næzæde budænd]

*Maxim of relation*

In the maxim of relation, it is emphasized to mention this point and that don’t say irrelevant speech [26].

- Example 1: [koronɑ dær kæmin ʔæst / mɑsk rɑ færɑmuʃ nækonid]
- Example 2: [mɑsk e mæn ʔæz to mohɑfezæt mikonæd/mɑsk e to ʔæz mæn mohɑfezæt mikonæd]

*Maxim of manner*

In the maxim of manner, attention is paid to four principles. First, avoid obscurity in speech. Second, do not use ambiguity in speech. Thirdly, the speech should be summary and avoid unnecessary speech. And fourth, the speech should be orderly and regular [26].

- Example 1: [moj e jædid e koronɑ dær rɑh ʔæst hær che særiʔtær vɑksæn bezænid]
- Example 2: [vɑksæn, ʔærzɑntærin væ behtærin ræveʃ e mohɑfezæt ʔæz χod væ ʔæzizɑnemɑn]

**Using the pronouns “we” and “you”**

The use of the pronoun “we” shows that the producer of the text considers her/his audience a part of herself/himself and establishes a closer relationship with them [26]. Meanwhile, the use of the pronoun “you” indicates that the producer of the text considers herself/himself separate from the audience [26].

- Example 1: [mɑ ʃirɑzihɑ ghæbl ʔæz χoruj ʔæz mænzel mɑsk mizænim. mɑ mɑsk mizænim]
- Example 2: [mɑsk rɑ færɑmuʃ nækonid]

##### Expressive value

*Indicator characteristics of expressive methods*

Frequent use of the imperative, simple and active sentences are among the most frequently used features of the expression methods in advertising texts [38]. The imperative mood is actually used to express advertising that somehow wants the audience to do something directly [48]. The repeated expression of the content using simple and active sentences also shows that the producer of the text provides the audience with information that has more than enough evidence to confirm them [38]. Also, using this type of text expression is actually providing an incentive tool to attract the attention of the audience [38]. Examples related to imperative mood, simple and active sentences are mentioned in the previous titles.

#### Cohesion analysis

##### Reference

Reference is traditionally used in semantics for the connection between a word and what that word refers to in the real world [52]. The most common reference categories in Persian and most languages are pronouns [52].

- Example 1: [næghʃe **χodæt** rɑ dær sælɑmæt ʔifɑ kon…
- mɑsk bepuʃim
- mɑsk e **mæn** ʔæz **to** mohɑfezæt mikonæd/mɑsk e **to** ʔæz **mæn** mohɑfezæt mikonæd]

##### Substitution

In substitution one word (or several words), it replaces another word (or words) [52].

- Example 1: [Mæn bærɑye **hefz e sælɑmæt** e to mɑsk mizænæm / to hæm bærɑye **sælɑmæt** e mæn **lotfæn** mɑsk bezæn]

##### Conjunction

Conjunction sentences are made of two or more independent sentences that are connected by conjunction words [52]. These words are: but, or, and, that, if, because, until, unless, because, both… both, what… what, or… or, not… not, whether … whether, not only … but etc. [52].

- Example 1: [**vɑksæn bezænid tɑ zende bemɑnid**]

##### Lexical cohesion

Lexical coherence investigates the role played by the choice of words in organizing relationships within a text [52]. Lexical continuity is divided into two major categories: reiteration and collocation.

**Reiteration**

Reiteration means repeating a word in such a way that it can add to the inner music and increase the effect of the speech [52].

- Example 1: [mɑsk e mæn ʔæz to **mohɑfezæt mikonæd** / mɑsk e to ʔæz mæn **mohɑfezæt mikonæd**]

**Collocation**

Collocation means the occurrence of two or more words with a short distance within a corpus language, the choice of each word affects the choice of other words and their occurrence [52]. In the example below, the words “handkerchief”, “gloves” and “mask” are part of the set of words that were emphasized on their use during the Corona virus.

- Example 1: [**dæstmɑl**, **dæstkeʃ** væ **mɑsk** e χod rɑ pæs ʔæz ʔetefɑde dær sætl e zobɑle dærbdɑr biyændɑzim]

#### Text structure analysis

##### Emphasis

That part of the sentence that has new information is called emphasis. The emphasis expresses the salient and emphasized points of the author and close the way to an open talk [52]. Emphasis has marked emphasis and unmarked emphasis types. The structure that is close to the verb has unmarked emphasis [52]. In Persian language, the structure closest to the verb is the object. Marked emphasis is also a type of emphasis that, in addition to having new information, the emphasized structure selects an element of a set and contrasts it with other members and has a contrasting meaning [52]. In Persian language, cleft and pseudo-cleft sentences have marked emphasis.

*Marked emphasis:*

- Example 1: [mɑ ʃirɑzihɑ **tɑ ʃekæst e koronɑ** hæmegi **ʔæz mɑsk** ʔestefɑde mikonim]

*Unmarked emphasis:*

- Example 1: [**mɑsk rɑ** færɑmuʃ nækonid]

##### Interaction solutions

**Attitude markers**

Attitude markers are words and phrases that are used in the text and communicate directly with the audience [52]. Such as “note that”, “see”, “pay attention”, “be careful” etc. [52].

- Example 1: [**væzʔiyæt e ghermez** / **morɑgheb bɑʃim** tɑ pæʃimɑn næʃævim]

**Engagement markers**

Engagement markers mean the author’s opinion and attitude towards the topic under discussion [52]. Some of the words or phrases that show the author’s opinion and attitude are: “fortunately”, “happily”, “unfortunately” etc. [52].

- Example 1: [mæn ʔæz ʔɑn pezeʃk væ pæræstɑr vɑgheʔæn χejɑlæt mikeʃæm ke ʔɑnhɑ ʔɑnjur dɑrænd fædɑkɑri mikonænd]

### Statistical analysis

In this section, people’s attitude towards linguistic components is investigated using a survey that was distributed an online questionnaire, so that based on the highest frequency, the discourse pattern of medical advertisements can be discovered and compiled. This study was conducted with the participation of 350 Iranian adults. The average age of the participants was41.94%. 199 of the participants (56.86%) were female and 151 (43.14%) were male. 224 (64%) were married, 95 people (27.14%) were single, 12 people (3.43%) were divorced and 19 people (5.43%) were widows. 48 people (13.71%) have free jobs, 59 people (16.86%) have government jobs, 15 people (4.29%) workers, 117 people (33.43%) were students, 24 people (6.86%) are housewives, 54 people (15.43%) are retired, 14 people (4%) were unemployed and 19 people (5.42%) had other jobs. Also, the number of 12 people (3.43%) low education, 40 people (11.43%) diploma, 114 people (32.57%) were bachelor’s degree, 83 people (23.71%) were master’s degree, 63 people (18%) were PhD and 38 people (10.86%) were postdoctoral.

Table 3 shows the results of the descriptive statistics analysis of the audience’s attitude towards linguistic components. The analysis of the data in Table 3 shows that the opinion of the audience regarding the types of words is as follows: in the case of simple words, 59.43% of people chose the medium option and 4% of people chose the very low option; regarding compound words, 46.86% chose much option and 4.29% chose very much option; In relation to derivative words, 56.57% of option are very much and 2.29% are very low option, and in relation to derivative-compound words, 30.57% chose the medium option and 6.86% chose the very low option, all of which included the highest and lowest amount, respectively. In relation to the words that are used with a special ideology, the analysis also shows that 36.86% of people chose the high option and 5.14% of the people chose the very low option, which is the highest and lowest average, respectively. Regarding the synonym words, 79.71% of the option were very much and 0/86%were very low option; in relation to opposite words, 73.14% of option were much and 2.86% were very few option, in relation to official words, 64.86% were many option and 2.86% were very low option; informal vocabulary, 42% low choice and 0/57% very low choice; and in relation to the vocabulary conceptual signification, 40.29% chose the much option and 4.86% chose the very low option, which were the highest and lowest respectively. Also, the survey about living metaphors shows that 68.57% of people choose very much option and 0/86% have chosen very low option. Regarding dead metaphors, 54.57% of the option are very much and 2.29% were very low and regarding allusion, 51.43% have chosen very much option and 0/29% have chosen very low option, which include the highest and lowest number, respectively. The survey about the scrambling distance short shows that 39.14% of people chose the option very much and 2.29% chose the option very low; regarding the scrambling distance long, 38.29% of option were very much and 4% were very low; in relation to semantic ellipsis, 50.29% of option were very much, 0/86% were very low option; in the case of verbal ellipsis, 44.28% have chosen the very much option and 2% have chosen the very low option, all of which include the highest and lowest averages, respectively. Regarding the types of semantic roles, the highest and lowest averages were as follows: agent: 35.14% much option and 2.29% very low option; experiencer: 37.43% much option and 5.14% very low option; stimulus: 40.57% medium option and 6.57% very low option; theme: 36% medium option and 11.14% very low option; patient: 53.14% much option and 2.29% very low option; instrument: 43.14% very much option and 3.15% very low option; location: 68.29% much option and 0/29% very low option; direction or goal: 44.57% much option and 6% very low option; recipient: 51.14% medium option and 10% very low option; source or origin: 36.86% much option and 3.15% very low option; tense: 63.14% many option and 2% very few option; beneficiary: 70.57% much option and 1.14% very low option; manner: 52.57% medium option and 5.43% very low option; purpose: 36.86% medium option and 4% very low option; regarding the cause: 30.29% of people chose the medium option and 6.57% chose the very low option. In the survey of active verb, 77.14% of the audience chose the very much option and 0/29% chose the very low option and in relation to topicalization, 69.14% chose very much option and 0/29% chose very low option, which were the highest and lowest average respectively. Regarding the types of mood, the highest and lowest averages were as follows: subjunctive: 46.86% high option and 6% very low option; imperative: 54% medium option and 4.86% very high option; indicative: 59.14% chose the high option and 2% chose the very low option. In relation to the types of important characteristics of the relation method, the highest and lowest averages were as follows: maxim of quantity: 79.71% very much option and 0/29% very low option; maxim of quality: 75.43% very much option and 0/29% very low option; maxim of relation: 83.14% very much option and 0/29% very low option; maxim of manner: 78.86% chose the very much option and 0/57% chose the very low option. Also, surveys about the pronoun “we” show that 86.29% of people chose the very much option and 0/29% of the people chose the very low option. and regarding the pronoun “you”, 84.57% chose the medium option and 0/86% chose the very much option. Also, in relation to expressive value, 42.29% chose the much option and 4.29% chose the very low option, which were the highest and lowest averages, respectively. The highest and lowest averages from the audience’s point of view regarding the types of coherence were as follows: reference: 50.86% very much option, 0/57% very low option; substitution: 35.43% very much option and 7.43% very low option; conjunction: 40.29% of much option and 6.86% of very low option; reiteration: 48.29% much option and 2.57% very low option; collocation: 50.86% very much option and 1.71% very low option. Regarding the types of text structures, the highest and lowest averages were as follows: marked emphasis: 44.86% very much option and 3.43% very low option; unmarked emphasis: 51.14% low option and 2.29% very much option; attitude markers: 62% very much option and 0/57% very low option; engagement markers: 54.57% chose much option and 1.43% chose the very low option.

**Table 3:** The frequency of people’s attitude towards linguistic components

Linguistic components					Attitudes									
					Very Much		Much		Medium		Low		Very Low	
					frequency	percentage	frequency	percentage	frequency	percentage	frequency	percentage	frequency	percentage
Words	Experimental Value	Lexical Class of Words	Simple Words		34	9.71	72	20.57	208	59.43	22	6.29	14	4
			Compound Words		15	4.29	164	46.86	98	28	41	11.71	32	9.15
			Derivational Words		93	26.57	198	56.57	32	9.15	19	5.43	8	2.29
			Derivative-Compound Words		72	20.57	86	24.57	107	30.57	61	17.43	24	6.86
		Vocabulary Ideology			106	30.29	129	36.86	66	18.86	31	8.86	18	5.14
		Synonym			21	6	279	79.71	41	11.71	6	1.71	3	0.86
		Opposite			32	9.14	256	73.14	37	10.57	17	4.86	8	2.29
	Relational Value	Formal Words			52	14.86	227	64.86	45	12.86	16	4.57	10	2.86
		Informal Words			2	0.57	68	19.43	92	26.29	147	42	41	11.71
	Expressive Value	Vocabulary Conceptual Signification			74	21.14	141	40.29	92	26.29	26	7.43	17	4.86
	Metaphor	Live Metaphor			240	68.57	75	21.43	28	8	4	1.14	3	0.86
		Dead Metaphor			191	54.57	99	28.29	39	11.14	13	3.71	8	2.29
	Allusion				180	51.43	165	47.14	3	0.86	1	0.29	1	0.29
Syntax	Experimental Value	Syntax Processes	Scrambling	Distance Short	137	39.14	127	36.29	46	13.14	32	9.14	8	2.29
				Distance Long	134	38.29	113	32.29	64	18.28	25	7.14	14	4
			Structural Deletion	Semantic Ellipsis	176	50.29	131	37.43	33	9.43	7	2	3	0.86
				Verbal Ellipsis	155	44.28	127	36.29	47	13.43	14	4	7	2
		Semantic Roles	Agent		118	33.71	123	35.14	74	21.14	27	7.72	8	2.29
			Experiencer		68	19.43	131	37.43	94	26.86	39	11.14	18	5.14
			Stimulus		52	14.86	87	24.86	142	40.57	46	13.14	23	6.57
			Theme		49	14	74	21.14	126	36	62	17.72	39	11.14
			Patient		99	28.29	186	53.14	42	12	15	4.29	8	2.29
			Instrument		151	43.14	132	37.71	37	10.57	19	5.43	11	3.15
			Location		58	16.57	239	68.29	49	14	3	0.86	1	0.29
			Direction or Goal		47	13.43	156	44.57	84	24	42	12	21	6
			Recipient		46	13.14	49	14	179	51.14	41	11.71	35	10
			Source or Origin		71	20.29	129	36.86	103	29.43	36	10.29	11	3.15
			Tense		67	19.14	221	63.14	38	10.86	17	4.86	7	2
			Beneficiary		53	15.14	247	70.57	39	11.14	7	2	4	1.14
			Manner		48	13.71	65	18.57	184	52.57	34	9.72	19	5.43
			Purpose		64	18.28	102	29.14	129	36.86	41	11.71	14	4
			Cause		74	21.14	96	27.43	106	30.29	51	14.57	23	6.57
		Topicalization			242	69.14	83	23.71	23	6.57	1	0.29	1	0.29
		Active Verb			270	77.14	53	15.14	24	6.86	2	0.57	1	0.29
	Relational Value	Mood	Subjunctive		43	12.29	164	46.86	94	26.86	28	8	21	6
			Imperative		17	4.86	47	13.43	189	54	74	21.14	23	6.57
			Indicative		74	21.14	207	59.14	44	12.57	18	5.14	7	2
		Important Characteristics of the Relation Method	Maxim of Quantity		279	79.71	49	14	19	5.43	2	0.57	1	0.29
			Maxim of Quality		264	75.43	67	19.14	15	4.29	3	0.86	1	0.29
			Maxim of Relation		291	83.14	39	11.14	14	4	4	1.14	2	0.57
			Maxim of Manner		276	78.86	47	13.43	18	5.14	7	2	2	0.57
		“we” Pronoun			302	86.29	29	8.29	17	4.86	1	0.20	1	0.29
		“you” Pronoun			3	0.86	37	10.57	296	84.57	9	2.57	5	1.43
	Expressive Value	Indicator Characteristics of Expressive Methods			86	24.57	148	42.29	69	19.71	32	9.14	15	4.29
Coherence	Reference				178	50.86	132	37.71	34	9.71	4	1.14	2	0.57
	Substitution				67	19.14	124	35.43	95	27.14	38	10.86	26	7.43
	Conjunction				65	18.57	141	40.29	74	21.14	46	13.14	24	6.86
	Lexical Cohesion	Reiteration			106	30.29	169	48.29	51	14.57	15	4.29	9	2.57
		Collocation			178	50.86	117	33.43	32	9.14	17	4.86	6	1.71
Text Structure	Emphasis	Marked Emphasis			157	44.86	128	36.57	34	9.71	19	5.43	12	3.43
		Unmarked Emphasis			8	2.29	13	3.71	93	26.57	179	51.14	57	16.29
	Interaction Solutions	Attitude Markers			217	62	104	29.71	19	5.43	8	2.29	2	0.57
		Engagement Markers			79	22.57	191	54.57	61	17.43	14	4	5	1.43

Based on qualitative data analysis as well as statistical analysis of people’s attitudes in Table 3, the core and main layers of the discourse pattern of medical advertisements related to the promotion of preventive behaviors and vaccination of the corona pandemic were identified and classified based on the intensity of impact on the audience and the level of attraction of people. Then, the classes that needed further development were corrected and finally, the researchers created the final framework of the paradigmatic model in the field of medical discourse advertising. In Table 4, the final and organized model is presented.

**Table 4:** The final and organized model

Words Pattern	Syntax Pattern	Coherence Pattern	Text Structure Pattern
Live Metaphor Dead Metaphor Allusion Synonym Opposite Formal Words Derivational Words Compound Words Vocabulary Ideology	“We” Pronoun Maxim of Relation Maxim of Quantity Maxim of Manner Active Verb Maxim of Quality Topicalization Instrument Beneficiary Tense Indicative Patient Subjunctive Direction or Goal Indicator Characteristics of Expressive Methods Experiencer Source or Origin Agent	Collocation / Reference Reiteration Conjunction Substitution	Attitude Markers Marked Emphasis Engagement Markers

## Discussion

The purpose of this research was to analyze the discourse structures of infographics and advertising billboards related to promoting preventive behaviors and taking measures to vaccinate the corona pandemic in the general adult population of Iran. Based on this, the components related to each linguistic title in the order of priority and frequency are as follows: ideal elements related to the lexical pattern include “Live Metaphor, Dead Metaphor, Allusion, Synonym, Opposite, Formal Words, Derivational Words, Compound Words, Vocabulary Ideology”, ideal elements related to the syntax pattern include ““We” Pronoun, Maxim of Relation, Maxim of Quantity, Maxim of Manner, Active Verb, Maxim of Quality, Topicalization, Instrument, Beneficiary, Tense, Indicative, Patient, Subjunctive, Direction or Goal, Indicator Characteristics of Expressive Methods, Experiencer, Source or Origin, Agent”, ideal elements related to the coherence pattern include “Collocation / Reference, Reiteration, Conjunction, Substitution”, ideal elements related to the text structure pattern include “Attitude Markers, Marked Emphasis, Engagement Markers”. Among these, the most key, ideal and basic language pattern is this pattern: [Words Pattern: Live Metaphor/ Syntax Pattern: “We” Pronoun/ Coherence Pattern: Collocation & Reference/ Text Structure Pattern: Attitude Markers]. Thus, the results of this study, in line with previous studies, showed that the language of health education and effective health communication, especially during an epidemic such as Corona, the more it is consistent with the way of thinking and expression pattern of ordinary people, the better, faster and more effective it will be possible to transmit messages [28–31]. What is important in this context is to identify these effective lexical and linguistic patterns in order to use them as best as possible.

Accordingly, according to the findings of this research, advertising texts that use “living metaphor” and “dead metaphor” have had the greatest impact on the audience. In this context, it can be argued that metaphors are concepts that refer to the understanding of an unknown conceptual domain based on another known conceptual domain. Because of this, it made it easy to understand the concept of Corona, which was very difficult for ordinary people at first. Among these, living metaphors are more attractive and have attracted more audiences because they are not yet in general use and have not been recorded in the vocabulary lists of language speakers. This argument, in addition to being in accordance with the theoretical foundations and methodological approach of the current research [35], is in line with the research of Cliff and Anna [28]. Also, the data analysis shows that the use of living metaphors indicates language changes. This problem is a natural thing and it occurs due to environmental changes and the needs of people in the society. This result is in line with the researches of Mweri [29], Anyanwu and Udoh [30] and Katermina and Lipiridi [31]. Another dimension that was examined in this research was the synonym and opposite. Based on the findings of the present research, texts that use synonyms and opposite words attract more audiences. In this context, it can be argued that when words with similar and opposite meanings are placed in a text, they present the intended concept with more emphasis to the audience. This emphasis shows the importance of the desired topic and as a result, it encourages the audience to perform the requested activity. This argument is a confirmation of the theoretical foundations and methodological approach of the current research [35]. Formal and informal words was another component that was investigated. The findings indicate that in this regard, texts that have used official words in their expression style have attracted more audiences. This issue is based on the induction of valuing, importance and respect to the audience, which is especially evident in Iranian culture, which is based on compliments, and in which there are many lexical distinctions to pay respect to the other person in different situations. As in the present study, this finding was also obtained. Another component that was investigated, is the vocabulary structure (including simple words, derivational, compound and compound-derivation). Among them, derivational words and compound words have attracted more audiences due to the simplicity in understanding the meaning and the marked of the word. This argument is in accordance with the theoretical foundations and methodological approach of the current research [35]. Also, another dimension that was investigated is the vocabulary ideology. Based on the findings of the present research, the texts in which content highlighting is placed on the main word related to the strategy of prevention or coping with the disease have attracted more audiences. In most languages of the world and especially in Persian, the words that are highlighted usually have a specific ideology. Also, the words that the text producer wants to be the center of the audience’s attention and convey the information message in the best possible way, are usually placed at the beginning of the sentence. This issue is also aligned with the theoretical basis related to the ideology of words [44].

Based on the findings of the present research, the analysis of the syntactic structure of the sentences shows that the use of the pronoun “we” in advertising texts has the greatest impact on the audience. In this regard, it is argued that the use of the pronoun “we” indicates that the producer of the text considers the audience as a part of herself/himself and in this way tries to establish a friendly and intimate relationship with the audience. Therefore, persuading people to perform preventive behaviors is not achieved only by providing medical information. Rather, it should be through the integration of preventive information along with the increase of motivational linguistic factors in order to be able to attract the audience more. In addition to being in line with the theoretical foundations of the present research, these results are also in line with the research of Fayyaz and Satti [33]. Also, in order to investigate the syntactic structure more deeply, the important characteristics of the communication method were analyzed and investigated. The results showed that when communicating effectively, the four principles of “maxim of relation”, “maxim of quantity”, “maxim of manner” and “maxim of quality” should be observed so that more audiences are attracted to the message. In this context, it is argued that speaking clearly and fluently, avoiding ambiguity and providing correct information, will make the audience understand the message correctly. This result is consistent with Wardhaugh’s view [26]. The investigation and analysis of "semantic roles" shows that the components of “instrument”, “beneficiary”, “tense”, “patient” and “goal” had a great impact on attracting the audience. Considering that in the semantic roles, noun phrases play a role based on the action or state described by the main verb, it can be argued that its use can be understood as a collaborative concept by the audience and thus attract more audiences to the message. In the examination of active verbs and passive verbs, it was also found that active verbs have been more successful in attracting the audience due to the fact that its subject is known. Examining the topicalization component shows that the texts in which topicalization is used have attracted more audiences. This issue can be argued in this way that because the subject position is the position where important, new and emphasized information is placed, therefore, the audience unconsciously considers the information that is placed in this position important and pays special attention to it. This argument is also in accordance with the theoretical foundations and methodological approach of the research [52]. In the analysis of the mood, the indicative and the subjunctive have been able to instill a better expressive value to the audience in a way that has had a greater effect on the audience’s mind. In this regard, it can be explained that considering that the indicative expresses real events and honesty is one of the conditions for attracting the audience, the use of this component has been effective in attracting the audience. Also, the subjunctive has been used to express a request along with a please and wish, which is considered a form of politeness in Iranian culture, and the language reflects the culture of the people. Therefore, the audience is more encouraged to follow the prompts. This result is consistent with Wardhaugh’s view [26]. In the examination of the coherence pattern and the text structure pattern, it was also found that the use of the components of “collocation”, “reiteration”, “attitude markers” and “engagement markers” have attracted the most audience. Considering that the use of these components provides information and health tips to the audience in an emphatic and prominent way, therefore the audience understands the importance of the issue more and is encouraged to perform the desired behavior. This argument is in accordance with the theoretical foundations and methodological approach of the research [35].

## Conclusions

The results of this research show that linguistic factors such as the choice of words, grammar, coherence and textual structure in discourses related to medical advertisements have had a great impact on arousing public opinion in order to create a positive attitude towards preventive measures and vaccination during the Corona pandemic. In general, based on the results of this research, it can be concluded that linguistic factors are very effective in attracting the audience in order to persuade them to implement health messages. Therefore, when teaching health and preventive principles, the most important point is the correct and effective use of linguistic and discourse principles for maximum effectiveness. So that if an effective communication and verbal pattern is used and is in line with the way of thinking and speaking of the audience based on scientific principles, the main message will be transmitted faster, easier and more effectively. This issue becomes more apparent, especially during the outbreak of an epidemic (such as during the Corona epidemic) when universal and public preventive measures gain special value and importance. As the results of the present study also showed its importance in this regard. What is important is that there have been epidemics throughout human history, and the coronavirus will not be the first or the last. Therefore, conducting scientific research during the outbreak of this virus can be an opportunity to solve the challenges in this field. In general, as the results of the present study also showed, billboards and infographics are one of the most important advertising tools in the field of health due to their availability to all members of society. In this regard, scientific principles based on research can be used to increase attractiveness, the speed of message transmission and increase the number of audiences. As the present study also aimed to achieve the same principles. Thus, the findings and results of this research can help all public health officials and medical education centers to manage and control the crisis in a fully professional and planned manner in case of possible similar crises and communicate better with people about public health.

### Suggestions for future research

Using Fairclough’s three-dimensional discourse analysis model, this research has provided a new and systematic framework for the qualitative evaluation of the texts of billboards and infographics in the field of prevention of the coronavirus epidemic. Considering that this research is one of the first researches that tried to provide a practical model in the field of disease prevention advertisements, therefore, it is in the early stages and more investigations should be done based on the components presented in this field so that a global model in this field can be achieved based on it. Also, languages have principles and parameters. The principles exist in all languages and are universal, but the parameters are the linguistic differences that have caused a particular language to emerge and differ from one language to another. Languages reflect the culture of societies. Therefore, it is suggested that research in the field of disease prevention advertisements be conducted on the languages and cultures of different societies so that a comprehensive and complete model in this field can be obtained from the results of these studies. Also, in addition to the texts presented for the prevention of diseases, images are very important and effective element to attract the audience and encourage them to perform preventive behaviors, which if not designed and presented based on systematic principles, they could have the opposite result on the audience and cause irreparable damage in this field. Therefore, it is suggested that in future research, in addition to analyzing advertising texts in the field of medical science, especially in the field of disease prevention, researchers analyze the images related to advertising in this field in different languages and cultures.

## Data Availability

All data analyzed during this study are included in this published article.

## Abbreviation

COVID-19: Coronavirus disease
